# Precision imaging and evolving therapies in paragangliomas and pheochromocytomas: from molecular diagnostics to imaging-guided management

**DOI:** 10.1186/s13244-025-02195-z

**Published:** 2026-02-09

**Authors:** Aurelie Choucair, Anna Zdunek, Matthew Liao, Lisa Bodei, Desiree Deandreis, Jeeban Das, Remy Barbe, Emily Bergsland, Susan Geyer, Francois Bidault, Gabriel Garcia, Randy Yeh, Corinne Balleyguier, Nathalie Lassau, Laurent Dercle, Samy Ammari

**Affiliations:** 1 Department of Medical Imaging, Gustave Roussy Cancer Campus, University Paris-Saclay, 94805 Villejuif, France; 2 Biomaps, UMR1281 INSERM, CEA, CNRS, University Paris-Saclay, Institut Gustave Roussy, 94800 Villejuif, France; 3 Department of Medical Imaging, Saint Louis Hospital, Jounieh, Lebanon; 4 University of Pittsburgh School of Medicine, Pittsburgh, PA USA; 5 Columbia University Vagelos College of Physicians and Surgeons, New York, NY USA; 6 Departement of Nuclear Medicine, Memorial Sloan Kettering Cancer Center, New York, NY USA; 7 Departement of Radiology, Memorial Sloan Kettering Cancer Center, New York, NY USA; 8 UCSF Helen Diller Family Comprehensive Cancer Center, San Francisco, CA USA; 9 Department of Quantitative Health Sciences, Mayo Clinic, Rochester, MN 55905 USA; 10 Department of Radiology, New York Presbyterian Hospital, Columbia University Medical Center, New York, NY 10039 USA

**Keywords:** Paraganglioma, Pheochromocytoma, Computed tomography, Magnetic resonance imaging, Positron-emission tomography

## Abstract

**Abstract:**

Pheochromocytomas and paragangliomas (PPGLs) are rare neuroendocrine tumors originating from neural crest-derived chromaffin tissue, marked by clinical heterogeneity and substantial genetic underpinnings. With up to 70% of cases linked to germline or somatic mutations, including Succinate DeHydrogenase genetic alterations (SDHx), and Von Hippel-Lindau (VHL), genetic profiling is central to diagnosis, risk stratification, and therapeutic planning. Clinical presentation varies by tumor location and secretory status—from catecholamine-driven crises to mass effect in head and neck paragangliomas (H&N PGLs). The diagnostic workflow begins with biochemical testing, followed by high-resolution anatomical and functional imaging. Computed tomography (CT) and magnetic resonance imaging (MRI) remain essential for localization and staging, while radiopharmaceuticals such as ⁶⁸Ga-DOTA⁰-Tyr³-octreotate (⁶⁸Ga-DOTATATE), ¹⁸F-fluoro-L-dihydroxyphenylalanine (¹⁸F-FDOPA), and ¹³¹I-metaiodobenzylguanidine (¹³¹I-MIBG) refine tumor characterization and guide peptide receptor radiopharmaceutical therapy (RPT) with radiolabeled octreotide derivatives or therapeutic MIBG Imaging features such as size, necrosis, and diffusion restriction correlate with malignancy risk, but novel molecular imaging offer promise for more precise prognostication. Therapeutic options span from curative surgery to systemic therapies, including temozolomide, tyrosine kinase inhibitors, and nuclide therapy. Minimally invasive, image-guided interventions provide palliation for metastatic or inoperable disease. Importantly, artificial intelligence and molecular assays such as the NETest and ¹H-MRS are emerging as pivotal tools in real-time tumor monitoring, early relapse detection, and biomarker discovery. This review underscores the necessity of a multidisciplinary, genomics-informed, and imaging-guided approach to PPGL management. With the integration of advanced imaging and AI-driven analytics, precision oncology for PPGLs is transitioning from potential to practice.

**Critical relevance statement:**

This article offers an overview of the diverse manifestations of paragangliomas, illustrated with examples from various anatomical locations. It also highlights different patterns of tumor evolution and provides an up-to-date review of current management and therapeutic strategies, with a special focus on emerging AI-guided approaches.

**Key Points:**

- Review the genetic associations, including Von Hippel-Lindau, Multiple Endocrine Neoplasia, Neurofibromatosis, and Carney Triad.
- Overview of anatomical imaging features (CT and MRI) of paragangliomas.
- Improve knowledge about the different Nuclear Medicine and functional imaging techniques in detecting lesions, depending on their location, secretory function and underlying genetic mutation.
- Discuss the multiple radiopharmaceuticals available for Scintigraphy and PET-CT, according to the paraganglioma site and mutational pattern.

**Graphical Abstract:**

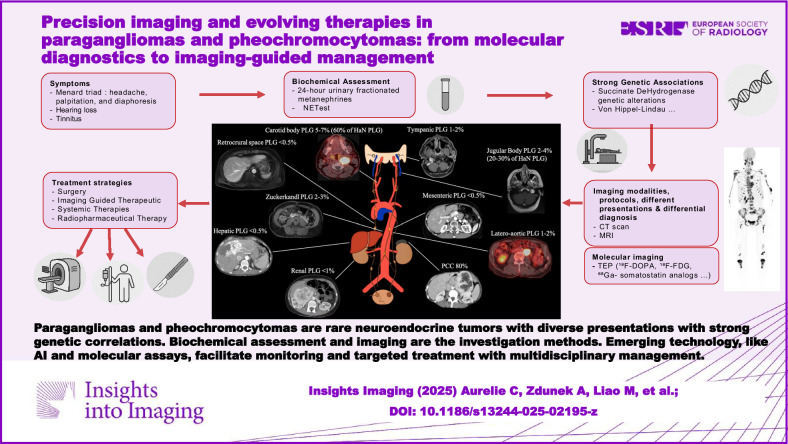

## Introduction

Pheochromocytomas (PCCs) and paragangliomas (PGLs), collectively referred to as PPGLs, are rare neuroendocrine tumors (NETs) with complex genetic backgrounds, diverse anatomical locations, and variable clinical behavior. Advances in genomics and functional imaging have revolutionized our understanding of these tumors, allowing for refined classification, risk stratification, and personalized management strategies. However, despite these advances, current clinical guidelines for PPGLs remain limited by the lack of consensus on optimal imaging strategies, radiotracer selection, and standardized diagnostic pathways.

This manuscript provides a comprehensive overview of PPGLs, beginning with their epidemiology, pathophysiology, and molecular genetics. We then review current diagnostic pathways, highlighting the role of biochemical assays and the expanding utility of anatomical and functional imaging--including CT, MRI, and nuclear medicine techniques. Special attention is given to imaging phenotypes and emerging biomarkers predictive of malignancy and metastatic potential.

The therapeutic landscape is explored in depth, from surgery to image-guided interventions, systemic therapies, and radiopharmaceutical therapy (RPT). We discuss the evolving role of artificial intelligence, radiomics, and molecular diagnostics in predicting treatment response and guiding follow-up. Finally, we examine future directions in precision oncology for PPGLs, emphasizing the need for multicenter data integration, advanced analytics, and biomarker-driven care.

## Clinical landscape and epidemiology

**Fig. 1:**
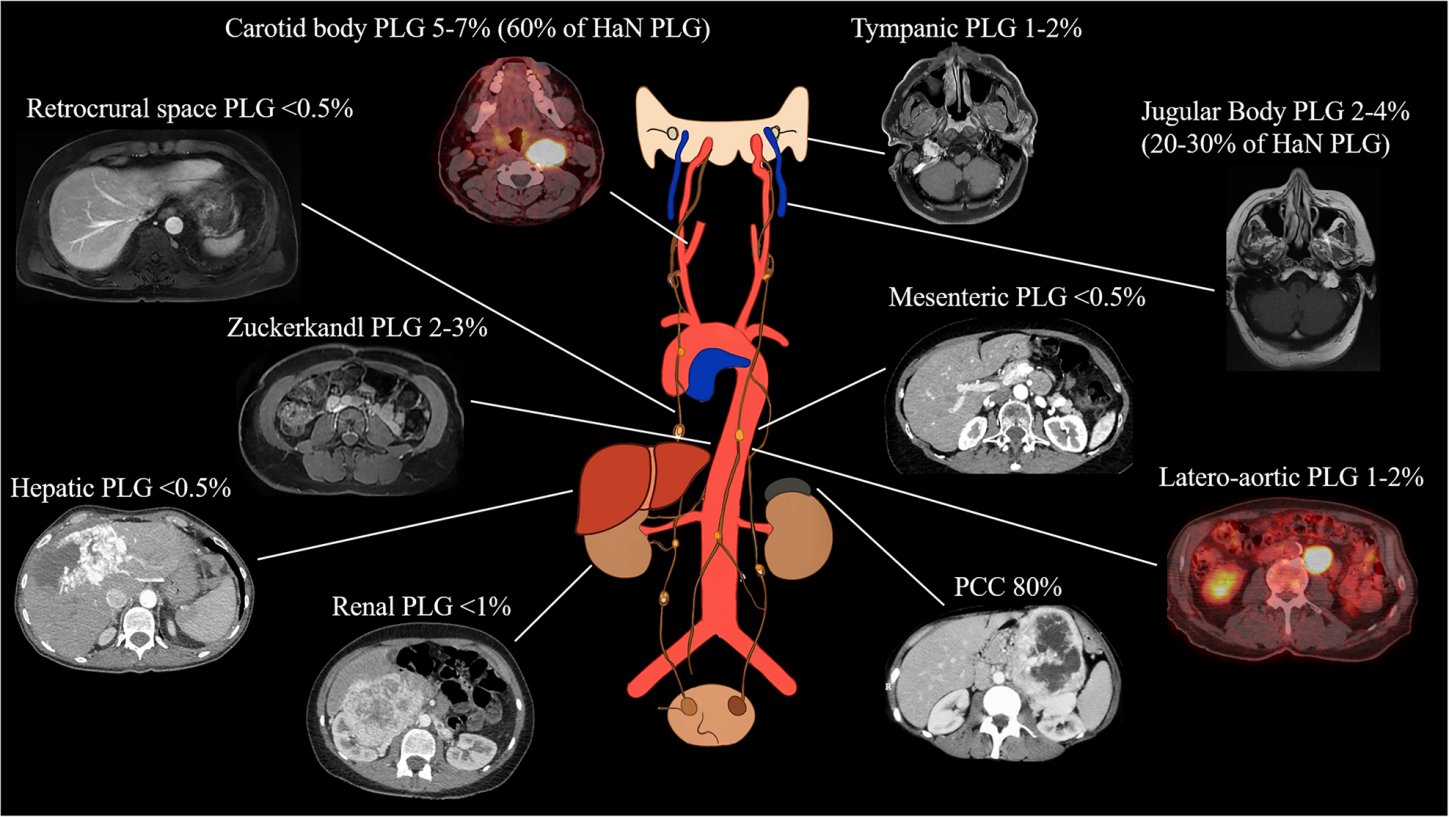
Frequency distribution of different PPLG locations

PPGLs are rare neuroendocrine tumors that are derived from neural crest cells. PCCs originate in the adrenal medulla, while PGLs arise from sympathetic (thoracoabdominal) or parasympathetic (head and neck). Together, PPGLs have an annual incidence of 2-8 per million, with PCCs comprising approximately 80% of cases (Fig. 1) [1]. These tumors contribute to 0.1%–0.6% of adults and 2%–4.5% of pediatric hypertension cases [1].

Among PPGLs, 10%–49% are incidentally discovered during imaging for other conditions, and 4%–8% of adrenal incidentalomas are PCCs [1]. Over recent decades, PPGL incidence has risen, primarily due to increased imaging utilization, biochemical testing, and genetic screening [2]. Tumors are now detected at earlier stages, with a smaller size and higher age at diagnosis [2].

PPGLs are classified as functional or non-functional based on catecholamine secretion. Approximately 96% of PCCs and 65% of PGLs secrete catecholamines—metanephrine, normetanephrine, and dopamine— leading to the classic *Menard Triad* of symptoms: headaches, palpitations, and diaphoresis [3, 4]. Other symptoms may include flushing, abdominal pain, diarrhea, nausea, and anxiety, as well as conditions like myocardial infarction, arrhythmias, and stroke in more severe cases [4].

In contrast, parasympathetic head and neck paragangliomas (H&N PGLs) are non-secretory in 95% of cases [5]. They typically present with local mass effect symptoms such as tinnitus, dysphonia, and hearing loss, and may be associated with Vernet, Collet-Sicard, and Horner syndromes due to compression of the cranial nerves [5, 6].

## Genetics

**Fig. 2:**
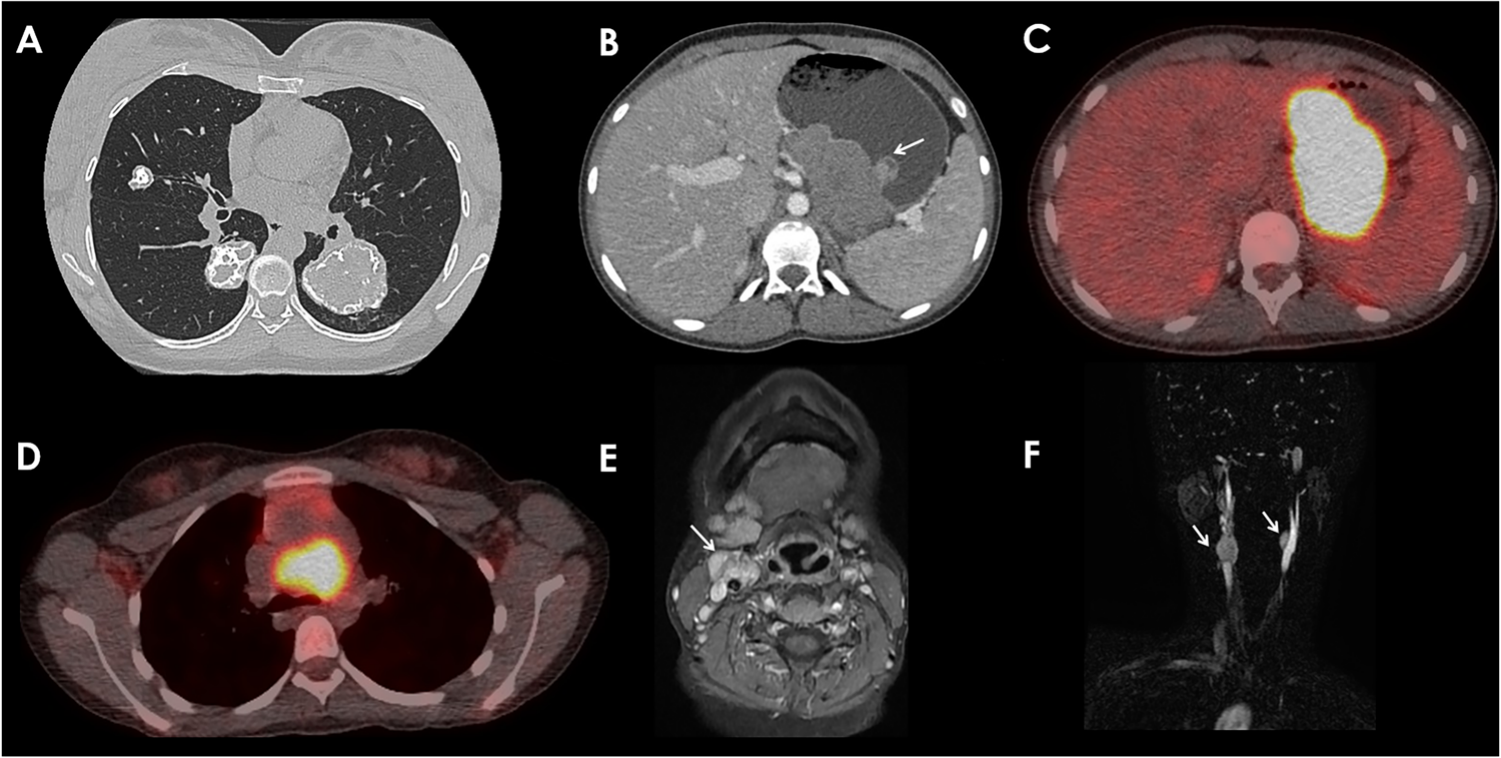
14 y.o. patient with SDHC Mutation presenting Carney Triad, which is a rare association of an extra-adrenal PGL, with pulmonary chondroma and a GIST. **A** Axial parenchyma CT Scan view of pulmonary chondroma, associated with a heterogeneous lobulated submucosal gastric mass corresponding to a mesenchymal tumors: GIST seen on the portal venous phase CT scan (**B**), high uptake at ^18^F-FDG PET-CT SUVmax 30.3 (**C**). In addition, there’s a mediastinal paraganglioma with a high uptake at 18F-FDG PET/CT SUV 10.2 (**D**), and cervical carotid body PGL: (**E**) T1-w CE MRI and Time-resolved imaging of contrast kinetics (Tricks®) MRA (**F**)

Genetics plays a crucial role in PPGL pathogenesis. Approximately 40% of PPGLs have a genetic basis, with over 20 genes identified [7]. Mutations in Succinate DeHydrogenase genetic alterations (SDHx) genes (SDHA, SDHB, SDHC, SDHD) account for the majority of cases, and clinical presentation varies between specific mutations [8]. For instance, SDHB mutations are linked to abdominal PGLs with high metastatic risk, while SDHD mutations more commonly result in benign, multifocal H&N PGLs [8, 9]. Other syndromes predisposing to PPGLs include Von Hippel-Lindau (VHL), Multiple Endocrine Neoplasia Type 2, and Neurofibromatosis Type 1 [10]. Carney Triad is a rare genetic association of PGL with pulmonary chondroma and Gastrointestinal Stromal tumor (GIST) (Fig. 2) [11].

Genomic profiling has led to molecular classification into four PPGL subtypes: kinase signaling, pseudohypoxia, Wnt-altered, and cortical admixture—each associated with distinct clinical behavior, familial risk, and metastatic potential [12]. The significant genetic component of PPGLs differentiates them from other NETs and necessitates genetic testing for diagnosis, prognosis, and management [3, 13], including in identifying an appropriate imaging modality. Thus, genetic screening is highly recommended as part of routine diagnostic algorithms [1].

## Diagnosis and biochemical testing

The initial diagnosis of PPGLs relies on biochemical testing. The preferred screening methods are plasma-free metanephrines or 24-h urinary fractionated metanephrines, due to their high sensitivity [4]. In selected cases, serum 3-methoxytyramine (for dopamine-secreting tumors) and chromogranin A may be used as supplemental markers [4].

Elevated metanephrine levels—especially values ≥ 4 times the upper limit of normal—strongly suggest PPGL and warrant anatomical imaging with CT or MRI for localization [4]. If levels are only mildly elevated, testing should be repeated to rule out confounding factors and may be followed by imaging to confirm disease presence and extent [4].

Biochemical profiles vary depending on tumor location and genetic mutations, reflecting differential secretion of catecholamines. Tissue biopsy is contraindicated in suspected PPGLs until catecholamine excess is ruled out, due to the risk of catecholamine crisis and severe hypertension [14]. Despite its importance, biochemical testing is prone to false results, which can result from physical activity prior to testing and medications like diuretics or beta-blockers [15]. In addition, 10% or more of PPGLs are biochemically silent, complicating the diagnosis [16, 17]. In such challenging cases, the neuroendocrine tumor (NET) test (NETest), a multigene blood-based test, can offer further diagnostic aid, testing tumor activity regardless of catecholamine production.

## Prognostic factors

PPGL malignancy is defined histologically only by the presence of distant metastases in non-chromaffin tissues, according to World Health Organization criteria [18]. Common metastatic sites include the bone, liver, lungs, lymph nodes, and abdomen/pelvis [19]. Metastatic risk is lower in PCCs (5%–10%) compared to PGLs (30%–35%), with the highest risk in SDHx mutation carriers [19, 20].

**Fig. 3:**
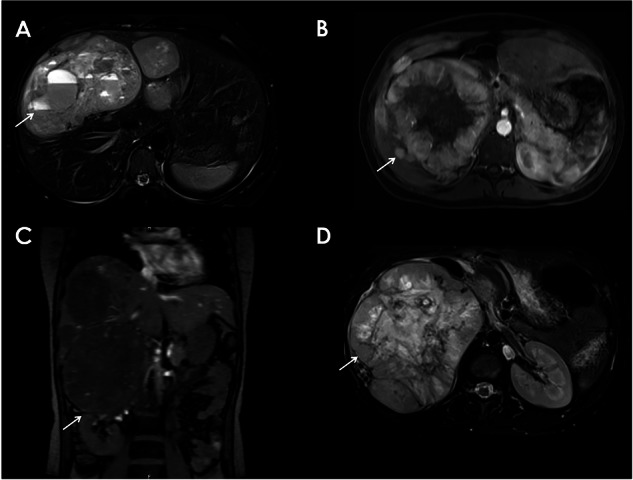
PCC with hepatic metastasis. Heterogeneous adrenal mass presenting a peripheral enhancement on T1-w CE MRI (**B**): central necrosis. We can see a downward displacement of the right kidney (**C**). Hepatic mass, with a cystic component hyperintense on T2-wMRI (**A**), with a heterogeneous enhancement on T1-w CE MRI (**D**)

The overall survival for malignant PPGLs ranges from 40% to 60%, with higher malignancy rates in PGLs than in PCCs [9]. Prognostic factors associated with worse outcomes include genotype, male sex, synchronous metastases (Fig. 3), larger tumor size (> 5 cm), extra-adrenal location, and an elevated serum dopamine metabolite 3-methoxytyramine [9, 20]. Ki-67, a marker of cellular proliferation, has also been shown to be a prognostic factor in PPGLs, particularly for metastatic PCCs [21–24].

Patient prognosis can be analyzed using various risk stratification tools. High scores using the histologic-based GAPP (Grading of Adrenal Pheochromocytoma and Paraganglioma) criteria [25] and clinical presentation-based ASES (Age, Size, Extra-adrenal location, and Secretory type) criteria [26] are negatively correlated with survival. Radiography further enhances risk stratification as imaging studies may reveal prognostic factors such as lesion size prior to surgical biopsy, and non-invasive techniques, such as MRI diffusion-weighted imaging (DWI) may assess cellularity similar to traditional histological analysis [27].

## Imaging and risk assessment in pheochromocytomas and paragangliomas (PPGLs)

### Anatomical imaging

Imaging plays a crucial role in diagnosing and managing patients with PPGLs. In particular, CT scans and MRI are the cornerstones of anatomical imaging for PPGLs. Radiographic studies are used for screening patients with a family history of PPGLs, confirming diagnoses, determining tumor location, staging, and assessing disease progression.

PPGLs on CT typically present as a dense mass with density > 10 Hounsfield units and high vascularity [28, 29]. Dual-phase contrast-enhanced (CE) CT helps differentiate PCCs from other adrenal masses as PCCs demonstrate < 50% washout after 10 min, although false negatives may occur with lipid-rich PCCs [30]. CT imaging also evaluates PPGL features, such as the early tumor “blush” seen 25 s post-contrast injection, which may appear homogeneous or heterogeneous due to necrosis, hemorrhage, or calcifications.

**Fig. 4:**
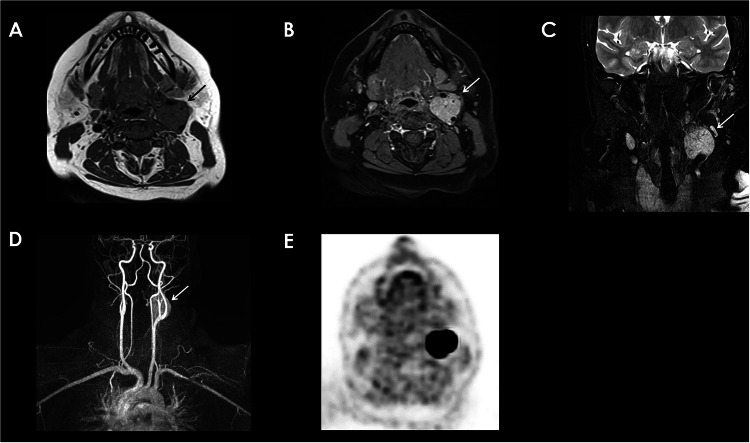
Left Carotid Body PGL. MRI (**A** to **D**): Well-circumscribed mass of intermediate signal intensity on Axial T1-w MR (A), high signal intensity on Coronal T2 MRI (**C**) was seen on the carotid bifurcation. It splays apart the internal (white dashed arrow) and external carotid (white arrow) arteries and expands the carotid bifurcation (**B**–**D**) “Lyre Sign”. The jugular vein is displaced posteriorly (**B**). It shows a “salt and pepper” appearance representing a combination of hemorrhagic regions/slow flow “salt” and regions of flow voids “pepper” (best seen on T2). After gadolinium administration, there is a vivid enhancement (**B**). Tricks® MRA sequence revealing the location of the PGL (**D**). High 68Ga-DOTA-SSTR uptake at PET-CT (**E**) SUVmax 58,8 (**E**)

On MRI, PPGLs exhibit strong and early contrast enhancement and appear hypointense on T1-weighted (T1-w) sequences while being markedly hyperintense on T2-weighted (T2-w) sequences [10]. A characteristic heterogeneous “salt and pepper” appearance may be observed, with the “salt” representing hemorrhagic components and the “pepper” representing rapid intratumoral vascular flows or necrosis, often suggesting cystic lesions or hemorrhagic necrosis (Fig. 4) [28, 31].

Imaging studies not only aid diagnosis but also provide prognostic insights preoperatively. Lesions > 5 cm are at greater risk of malignancy [20], and MRI DWI may offer a non-invasive alternative for assessing tumor cellularity, although further validation is required (Supplementary Fig. 2) [27].

#### Head and neck paragangliomas

**Fig. 5:**
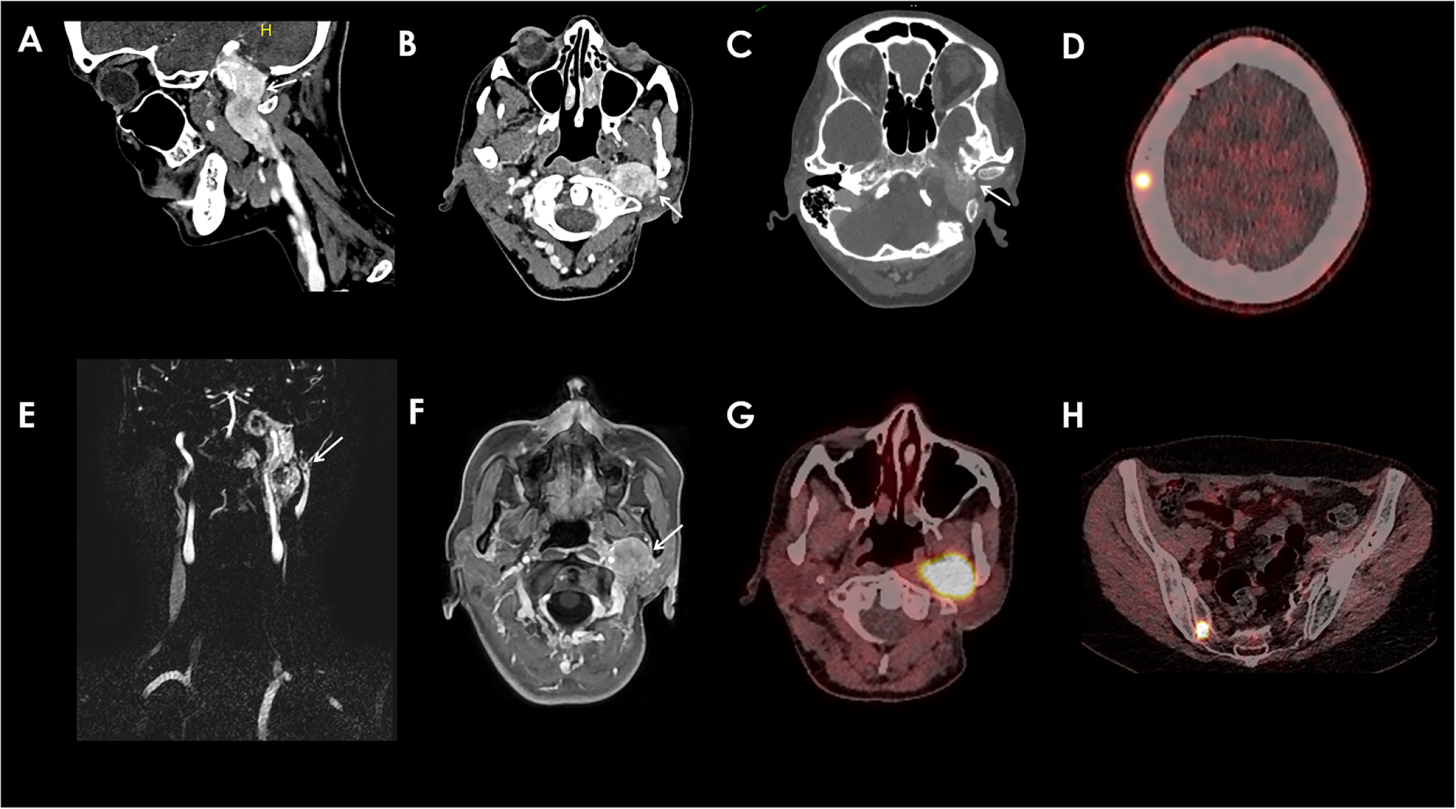
Jugulo-tympanic PGL. Lobulated mass located in the jugulo-tympanic space extending into the jugulo-carotid space, presenting a heterogeneous enhancement on the axial (**B**) and sagittal CT-Scans (**A**), measuring 42 × 52 mm, invading the hypoglossal canal, with an adjacent clivus lytic lesion (**C**). T1 Post Gadolinium Injection axial (**F**) and Tricks MRA sequence revealing the hypervascular aspect of the PGL (**E**). Increased 58 MBq de ^68^GA-DOTATOC uptake of the mass on the PET-CT (**G**) (SUVmax = 33,8). Lytic bone lesion of the skull (**D**) and the right iliac bone (**H**) with increased uptake on PET-CT (SUVmax = 41,34)

H&N PGLs are typically parasympathetic and non-secreting, with their anatomical features varying based on their location. The most common site for these tumors is the carotid bifurcation (Fig. 4), accounting for 60% of cases [5]. These tumors can compress the internal jugular vein and adjacent nerves, leading to clinical symptoms such as dysphagia, hoarseness, or Horner’s syndrome [10]. Tympanic PGL arises from the Jacobson nerve at the cochlear promontory and similarly causes hearing loss and tinnitus (Fig. 5) [10]. Jugular bulb tumors can involve both the jugular vein and carotid artery, with potential extension along the eustachian tube to the middle ear, causing symptoms such as hearing loss and tinnitus due to bone lysis [10]. Tympanic PGL, most commonly seen in women, arises from the Jacobson nerve at the cochlear promontory and similarly causes hearing loss and tinnitus [10]. Vagal PGL can compress the carotid bifurcation anteromedially and may involve the skull base, and these tumors are often asymptomatic but may present with dysphagia or vocal cord paralysis [10, 32]. Rarer forms of H&N PGLs include tracheal, laryngeal, and thyroid PGLs [33].

MRI is a critical tool for assessing H&N PGLs, with reported sensitivities of 90%–95% and specificities of 92%–99% due to its superior soft tissue contrast and ability to provide detailed anatomical and functional information [34, 35]. Typical workup for PGL includes unenhanced and enhanced fat-suppressed spin-echo T1-w and T2-w sequences [36]. Combining this imaging with CE three dimensional MR angiography (CE-MRA) further enhances diagnostic accuracy, as CE-MRA images a larger field of view and can detect smaller lesions than conventional MRI, making it useful for diagnosing multicentric PGLs (Figs. 4 and 5) [35, 36]. CE-MRA also reveals an early arterial enhancement, or tumor blush, which is unique to H&N PGLs compared to other H&N tumors. Dynamic CE (DCE) MRI and DWI are also particularly valuable in differentiating PGLs from other head and neck tumors such as schwannomas. DCE-MRI evaluates microvascular properties, revealing higher wash-in and wash-out rates in paragangliomas compared to tumors such as schwannomas [37, 38]. Additionally, lower apparent diffusion coefficient (ADC) on DWI can help distinguish H&N PGLs from other tumors and may correlate with the SDH mutation status of PGLs [37, 39].

Differential diagnoses for H&N PGLs vary based on their anatomical location: for masses located at the carotid body and jugular bulb lesions may mimic, it is essential to rule out lymphadenopathy, schwannomas, and neurofibromas [10]; lesions in the middle ear and petrous bone lesions can mimic conditions such as cholesterol granulomas, cholesteatomas, meningiomas, or other skull base tumors [40]. Imaging is crucial for the accurate assessment and differentiation of H&N PGLs from these other conditions.

#### Thoracic, abdominal, and pelvic paraganglioma

Thoracic, abdominal, and pelvic PGLs are catecholamine-secreting paragangliomas arising from chromaffin tissue [11]. When found in the adrenal gland, they are referred to as PCCs. These tumors can appear in various locations in the body, including along the sympathetic chain, within the organs of Zuckerkandl (Supplementary Fig. 1), and the posterior mediastinum (Supplementary Fig. 2), where differential diagnosis is hypervascular metastasis [41]. It can rarely develop in the liver (Supplementary Fig. 3) looking like an hepato-cellular carcinoma, in the kidney (Supplementary Fig. 4) mimicking a clear cell carcinoma and on the bladder wall [11]. These tumors present with symptoms of catecholamine excess, including the classic triad of headache, palpitations, and diaphoresis, as well as localized symptoms such as hematuria and micturition caused by bladder wall paragangliomas [11]. Tumors may also present with mass effects, such as chest pain and dyspnea associated with thoracic PGLs [42].

## Molecular imaging techniques

Molecular imaging provides critical information to enhance diagnostic precision, particularly in detecting metastases or characterizing lesions. Radiopharmaceuticals target specific receptors and metabolic processes, so the choice of radiopharmaceutical depends on the tumor’s location, secretory function, and genetic mutations [43, 44].

Molecular imaging techniques aid in diagnosing and monitoring PPGLs, each with specific advantages (Table 1) [45].

**Table 1:**
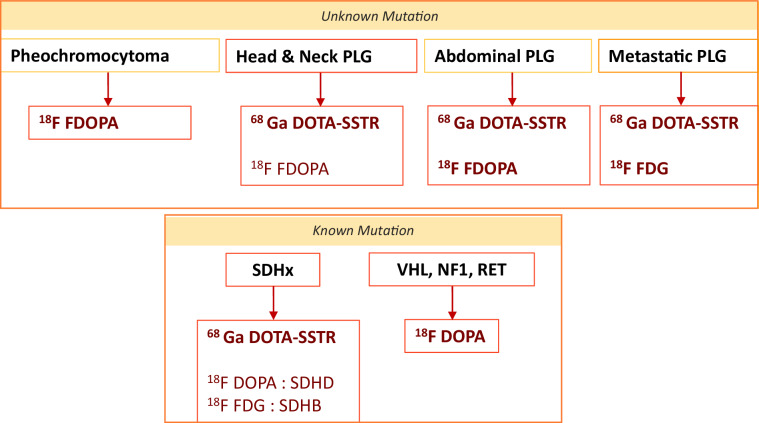
Functional imaging modalities according to paraganglioma location and patient mutational status

### Initial localization

For PCCs, Fluorine-18 fluoro dihydroxyphenylalanine (¹⁸F-FDOPA), a marker for dopamine synthesis, is preferred over ¹³¹I-metaiodobenzylguanidine (MIBG), a catecholamine analog in the detection of non-metastatic sporadic and inherited PCCs [43, 44]. Although MIBG was the gold standard and remains indicated for PCCs and abdominal PPGLs, it has lower specificity for thoracic and H&N PGL, and its limited resolution, reduced sensitivity for small lesions, and interference with physiological adrenal uptake make it primarily useful today for assessing eligibility for ¹³¹I-MIBG therapy (Supplementary Fig. 5) [46, 47]. On the other side, ¹⁸F-Fluorodopamine, known as ¹⁸F-FDA, has a low detection rate of 51.9%, and is less commonly used [48].

### Metastatic detection

Most PGLs and metastatic PPGLs (MPPGLs) are best imaged using ⁶⁴Cu- and ^68^Ga-DOTA-octreotide derivatives (DOTATOC and DOTATATE), which bind to somatostatin receptors (SSTRs) [44].

¹⁸F-FDG positron emission tomography PET is less effective for non-metastatic PCCs (58% sensitivity) but excels in the detection of metastatic and SDHx/VHL-related PPGLs (92%–99%) [44, 49, 50]. ¹⁸F-FDG PET-CT plays a greater role in the evaluation of rapidly growing or aggressive PGLs, particularly in cases of dedifferentiation, new metastatic lesions or rapid progression of existing metastases (Supplementary Fig. 6). However, there’s a lack of studies assessing its prognostic value across tracers. SUVmax is not yet standardized in clinical practice, and its interpretation depends on the radiopharmaceutical used. For instance, ^68^Ga-DOTATOC reflects SSTR expression, while the prognostic or biological significance of ¹⁸F-FDOPA and ¹⁸F-FDG uptake remains unclear, highlighting the need for cautious interpretation [51]. Incidental PGL detection may occur in the context of primary staging of a known malignancy, mimicking an FDG-avid metastatic lesion (Supplementary Fig. 7) [52].

## Therapy planning

Somatostatin receptor imaging is critical for therapy selection and monitoring. According to the European Association of Nuclear Medicine Guidelines, ⁶⁸Ga-somatostatin analogs (SSTA) are more specific for the detection and monitoring of PPGLs, particularly in metastatic, SDHB-related, sporadic and extra-adrenal disease [45]. Somatostatin antagonists, such as ⁶⁸Ga-NODAGA-JR11 and ⁶⁸Ga-LM3, induce stronger tumor uptake compared to the agonists, with high affinity to SSTR type 2, improving the evaluation [53].

Similarly, ¹⁷⁷Lutetium (¹⁷⁷Lu)-DOTA-JR11, a SSTR antagonist, induces better tumor uptake than ¹⁷⁷Lu-DOTATE, improving the image quality in RPT efficacy evaluation [53].

Future studies should focus on ¹⁸F-Silicon fluoride acceptor—lipophilic version—octreotate (¹⁸F-SiFAlinTATE), having a longer half-life, being cheaper, and having a tumor uptake comparable to ⁶⁸Ga-SSTA [54, 55].

### Therapeutic decision-making

Treatment of PPGLs is stage-dependent and individualized, with therapeutic decisions typically made within multidisciplinary tumor boards. The following overview summarizes the main treatment strategies according to disease stage, as recommended by the ESMO Clinical Practice Guidelines [56].

#### Localized disease

The ultimate PPGL treatment is surgery, whether it is a head-and-neck or a body lesion [57]. Preoperative imaging is essential to evaluate the size and extension of the tumor [44]. Abdominal PPGLs are treated with laparoscopy, except for large tumors where open surgery is necessary, especially when adjacent to vessels [58]. The mortality rate has decreased since the avoidance of catecholamine’s peak secretion, blood pressure and glycemia management [59]. Postoperative prevention of insulin release—as a response to catecholamine withdrawal—decreases hypoglycemia incidence [60]. Moreover, transarterial chemoembolization can be used preoperatively to decrease tumor size and alleviate surgical complications as arterial dissection [61, 62]. Annual follow-up is recommended as recurrence rates of PPGLs vary depending on tumor site, genotype, and prior therapy, and are estimated at around 1% in some series [63].

#### Metastatic disease

Tumor dissemination limits curative resection, although palliative and cytoreductive surgeries may be indicated in MPPGLs to improve the symptoms [56]. Multiple treatment modalities are proposed for disseminated PPGLs, as radiotherapy (RT), systemic and imaging-guided therapies [64].

##### Imaging-guided therapeutic options

Knowing that locoregional treatments are less invasive, they are used as palliative therapies to alleviate tumor size and decrease catecholamine secretion, reducing hypertension [65]. When complete resection isn’t an option, RT is suggested, especially for intra-thoracic and H&N PGLs, usually leading to fewer neurologic complications [66]. Moreover, MPPGLs can benefit from external beam radiation therapy (EBRT) to improve PGLs’ symptoms and reduce the tumor volume [67]. Metastasis and liver lesions in NETs can be treated by radiofrequency ablation, leading to tumor necrosis, with no contrast uptake on the next CT imaging [61]. Transarterial chemoembolization is also a suitable option to decrease tumor size within months [62]. In addition, polyvinyl or ethylene vinyl alcohol decreases blood flow in H&N PGLs, thereby stabilizing the tumor size. It can be a preoperative treatment in some cases, alleviating surgical complications as arterial dissection [68]. Moreover, few studies have shown that benign PCCs can be treated by imaging-guided percutaneous ethanol injection, leading to tumor necrosis [61]. Patients with risky bone metastases can benefit from percutaneous cementoplasty, osteosynthesis, and thermal ablation [69].

##### Systemic therapies: impact of new therapeutics in imaging management

Systemic therapies such as chemotherapies, targeted therapies, and immune inhibitors aim to halt the progression of the disease and control catecholamine secretion [70, 71]. Cytotoxic therapies such as the combination of cyclophosphamide, vincristine and dacarbazine contribute to catecholamine level control and PPGL size reduction of 30 to 70% [72]. Ayala-Ramirez et al showed a 6.4-year overall survival (OS) in metastatic patients priorly treated with chemotherapy, a progression-free survival (PFS) of 31 to 60 months, and a decreased tumor size and blood pressure in about 33% of patients [73, 74]. SDHB-mutated MPPGLs have shown benefit from temozolomide with prolonged PFS [75]. Nonetheless, targeted therapies, including tyrosine kinase inhibitors, offer prospects for enhancing PFS and decreasing PPGL size [76]. A randomized phase 2 study showed that 36% of metastatic patients benefit from sunitinib, with a 12-month PFS [76, 77]. Moreover, immunomodulatory agents such as interferon stimulate the immune system and promote cell growth inhibition [78]. In addition, cold SSTA has been approved as an anti-proliferative therapy in PGLs with high SSTR expression [20].

##### Radiopharmaceutical therapy

In nuclear medicine, peptide-conjugated radiotracers target specific receptors [76], leading to quick tissue integration and cell destruction [79]. Having a fast clearance, fewer side effects than chemotherapy, and being less expensive than immunotherapy, RPT is an efficient choice for MPPGLs [80].

High-specific-activity ¹³¹I-MIBG—even though inaccessible in many countries—was a Food and Drug Administration-approved therapy for MPPGL patients, alleviating the symptoms and extending the OS to 36.7 months [81]. When higher doses of ¹³¹I-MIBG were prescribed, the five-year OS improved, with more adverse effects, such as lung toxicity and myelosuppression [82, 83].

In general, RPT allows higher local doses with fewer side effects and a longer OS of approximately 6 years, with a PFS of 2.5 years [84].

¹⁷⁷Lu-DOTATATE (3rd-generation) has improved PFS in gastroenteropancreatic (GEP)-NETs, while ⁹⁰Y-DOTATOC is efficient for NETs in general [81, 85]. RPT is also an option for H&N PGLs when surgery or radiation aren’t possible [84].

New SSTR antagonist, ¹⁷⁷Lu-DOTA-JR11, induces better tumor uptake than ¹⁷⁷Lu-DOTATE, improving the RPT efficacy and reducing potential toxicities [53]. Patient eligibility depends on tracer uptake, risk factors, and treatment availability [81].

¹⁷⁷Lu-DOTATATE is effective in metastatic PPGLs, with biomarker response, but requires careful monitoring for hematologic toxicities; future validation is needed [86].

## Response evaluation

MPPGLs pose diagnostic and therapeutic challenges due to their heterogeneous evolution [87]. Initial imaging is crucial for evaluating disease stage and optimizing treatment strategies [44]. Changes in tumor morphology, devascularization, and increased ADC may indicate a treatment response, even when Response Evaluation Criteria in Solid Tumors (RECIST) criteria are not met [39]. In addition, treatment-related effects—such as hemorrhage or necrosis induced by antiangiogenic agents, or delayed responses following immunotherapy—further complicate response assessment [52, 88]. Owing to the limitations of RECIST 1.1, including lesion selection, inter-observer measurement, and delays in confirming a suspicion of progression, a correlation with metabolic imaging is crucial [52, 89]. PET-CT, molecular analysis and advanced segmentation are needed to evaluate the tumor burden and the treatment response [52]; however, specific standardized criteria for PPGLs are still lacking. Radiotracers targeting SSTR provide specific imaging, although their interpretation may be affected by physiological organ absorption and urinary excretion [90]. The SSTR-RADS 1.0 is highly reproducible and reliable for interpreting SSTR-PET-CT and guiding diagnosis and treatment in NETs. Nonetheless, it remains an emerging system requiring further validation in the PPGLs population [91, 92].

## Emerging technologies

The main limitation in developing reliable AI models for PPGLs lies in the requirement for large, well-annotated datasets—an ongoing challenge given the rarity and clinical heterogeneity of these tumors. Biomarkers play a crucial role in early tumor assessments, monitoring and detection of treatment response, guiding cost-effective therapies [64, 93]. Tumor Growth Rate may serve as an early predictor of progression in GEP-NETs, even in cases classified as stable by RECIST [94]. Machine learning-based analyses have demonstrated utility in distinguishing subclinical PCCs from lipid-poor adenomas and identifying malignant subtypes, supporting the potential application of AI in adrenal mass characterization [95].

While other imaging features, such as heterogeneity or ill-defined contours, have shown limited predictive value for malignancy [41], radiomics, which uses advanced imaging analytics, may improve risk stratification from imaging studies in the future, although further validation is necessary [80]. A recent multicenter study developed a CT-based radiomics and deep learning model that effectively predicts the metastatic potential of PPGLs and stratifies patients, highlighting the potential of AI-assisted imaging for individualized management [96]. Similarly, a deep learning-based AI model using ³⁶⁸Ga-DOTATATE PET-CT reliably automated the segmentation of metastatic PPGL lesions, enabling accurate calculation of tumor burden and potentially supporting objective therapy monitoring [97].

Magnetic Resonance Spectroscopy analyses different metabolites and helps characterize the tumors [98]. As an example, 1H-MRS SUCCES detects succinate and identifies SDHx mutations, which enables early diagnosis and helps in confirming the presence of metastases [99].

SSTA-RPT response and early disease progression have been predicted by the multigene circulating transcript biomarker NETest, reflecting real-time tumor activity, but it remains costly and needs randomized studies validation [100].

Bioinformatics has identified prognostic genes in PCCs, such as KCNH2, KCNQ2, KCNQ1 (good prognosis) and SCN2A (poor prognosis) [101]. Adding to that, some miRNAs, physiological gene regulators, are linked to worse prognosis and higher metastatic risk in PPGLs [102].

## Future research directions

Future research in PPGLs should be multicenter to overcome the rarity and heterogeneity of these tumors and to facilitate the development of AI models for diagnosis and response assessment. Advances in theranostics and radiopharmaceuticals will require clinical trials to assess safety, efficacy, and cost-effectiveness. Ultimately, collaboration between clinicians, imaging specialists, and scientists will be essential for personalized care of patients with PPGLs.

## Conclusion

PPGLs are no longer enigmatic tumors lurking in the shadows of incidental imaging. Once defined merely by location and catecholamine excess, these neuroendocrine neoplasms have emerged as molecularly complex entities requiring a precision-medicine lens. Today, their management demands more than anatomical localization—it necessitates an integrated approach that combines biochemical profiling, high-resolution imaging, genetic insights, and emerging digital tools.

Functional imaging, guided by genotype and secretory profile, now serves as a diagnostic compass, while therapies have expanded beyond the surgical suite to include targeted radionuclide treatments, systemic therapies, and minimally invasive interventions. The convergence of artificial intelligence, radiomics, and molecular diagnostics promises not only earlier detection but smarter, real-time treatment adaptations.

Yet, challenges remain. The rarity and heterogeneity of PPGLs continue to hinder large-scale validation of prognostic biomarkers. Access to novel radiopharmaceuticals is uneven. AI applications, while promising, require rigorous clinical integration and ethical oversight.

Looking ahead, the path is clear: a multidisciplinary model fueled by data, empowered by technology, and personalized through molecular understanding. Achieving this vision will depend on international collaboration and prospective studies. In this new era, precision imaging is not just a diagnostic tool; it is the foundation of predictive, preventive, and participatory care in neuroendocrine oncology.

## Supplementary information

ELECTRONIC SUPPLEMENTARY MATERIAL

## Data Availability

The datasets analyzed during this study are publicly available and appropriately cited throughout the manuscript. All data supporting the conclusions of this article are included within the published literature referenced in this work.

## Abbreviations

¹³¹I-MIBG Iodine: 131 Metaiodobenzylguanidine
¹⁷⁷Lu: Lutetium 177
¹⁸F: Fluorine 18
¹⁸F-FDA: ¹⁸F-Fluorodopamine
¹⁸F-FDG: ¹⁸F-Fluorodeoxyglucose
¹⁸F-FDOPA: ¹⁸F-Fluoro-L-dihydroxyphenylalanine
¹⁸F-SiFAlinTATE: ¹⁸F-Silicon-fluoride-acceptor–octreotate, lipophilic version
^68^Ga: Gallium 68
⁶⁴Cu: Copper 64
⁹⁰Y: Yttrium 90
ADC: Apparent diffusion coefficient
CE: Contrast enhanced
CT: Computed tomography
DCE: Dynamic contrast enhanced
DOTATATE: DOTA⁰-Tyr³-octreotate
DOTATOC: DOTA⁰-Phe¹-Tyr³-octreotide
DWI: Diffusion-weighted imaging
GEP: Gastroenteropancreatic
H&N PGL: Head and neck paraganglioma
MRA: Magnetic resonance angiography
MRI: Magnetic resonance imaging
NETs: Neuroendocrine tumors
PCC: Pheochromocytoma
PET: Positron emission tomography
PFS: Progression-free survival
PGLs: Paragangliomas
PPGLs: Pheochromocytomas and paragangliomas
RECIST: Response Evaluation Criteria in Solid Tumors
RPT: Radiopharmaceutical therapy
SDHx: Succinate DeHydrogenase genetic alterations
SSTA: Somatostatin analogs
SSTR: Somatostatin receptor
SUV: Standardized uptake value
T1-wor T2-w: T1-weighted or T2-weighted (MRI sequence)
Tricks: Time-resolved imaging of contrast kinetics
VHL: Von Hippel-Lindau
