# Supplementary material for: Precision imaging and evolving therapies in paragangliomas and pheochromocytomas: from molecular diagnostics to imaging-guided management
Source: Insights Imaging. 2026 Feb 9;17:37. doi: 10.1186/s13244-025-02195-z (PMC12886687; doi:10.1186/s13244-025-02195-z)

Precision Imaging and Evolving Therapies in Paragangliomas and Pheochromocytomas: From Molecular Diagnostics to Imaging-Guided Management

ELECTRONIC SUPPLEMENTARY MATERIAL

Supplementary Figure 1. PGL of Zuckerkandl organ.

MRI shows a well defined mass lying between the inferior vena cava and the abdominal aorta with high signal intensity on T2 signal (A) and heterogeneous enhancement on the T1-w CE acquisition (B).

^{123}I-MIBG : Norepinephrine analog, single photon emission CT showing high PLG uptake on the axial (C), coronal (D-E) and sagittal views (F).

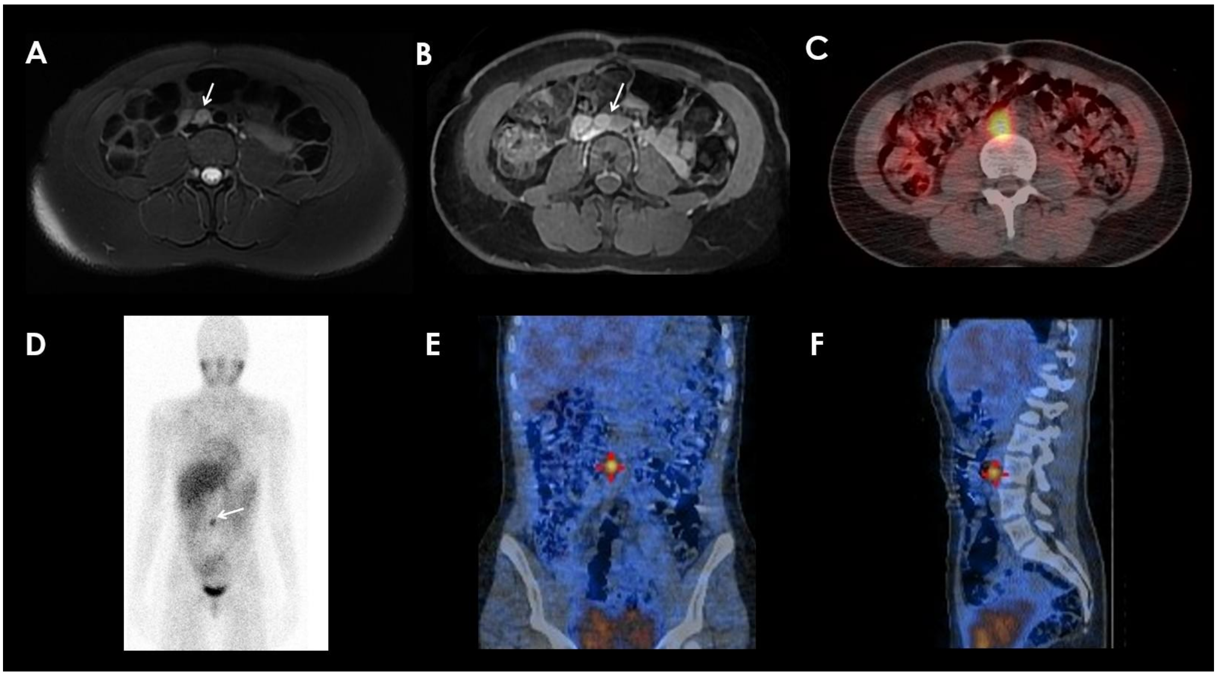

Supplementary Figure 2. Retrocrural space PGL (Postero-inferior Mediastinum): sympathetic nerve chain PGL.

64 y.o with SDHA mutation presenting a left paravertebral nodule, adjacent to T10, with a progressive homogenous enhancement on T1-w CE MRI (A), and restriction on DWI(C). It measures 12mm. There’s a discrete somatostatin uptake on PET-DOTA-SSTR (B).

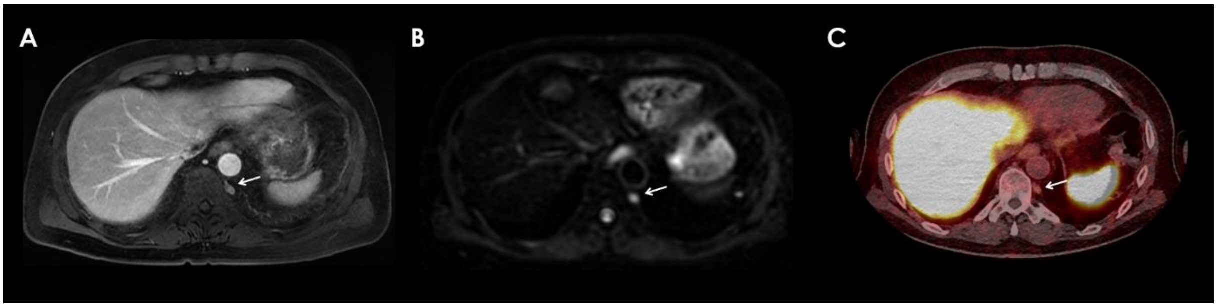

Supplementary Figure 3. Image of a primary malignant hepatic PGL, a rare extra-adrenal neuroendocrine tumor.

Unenhanced CT Scan (A), and contrast-enhanced arterial phase (B) showing a large hyper-enhanced, well-marginated sub-capsular well-marginated mass located in segment VI and VIII of the liver with areas of cystic necrosis inside (white arrow). The portal phase (C) shows a hepatic homogenization. T2-w MRI Fat Saturation (D) shows multiple serpiginous flow-voids inside. Avid enhancement was noted on T1-w CE MRI on arterial (E) and portal phase (F). High uptake at ^{18}F-DOPA PET-CT SUV max 12.1. A biopsy confirmed the diagnosis. It's a differential diagnosis of hepatocellular carcinoma.

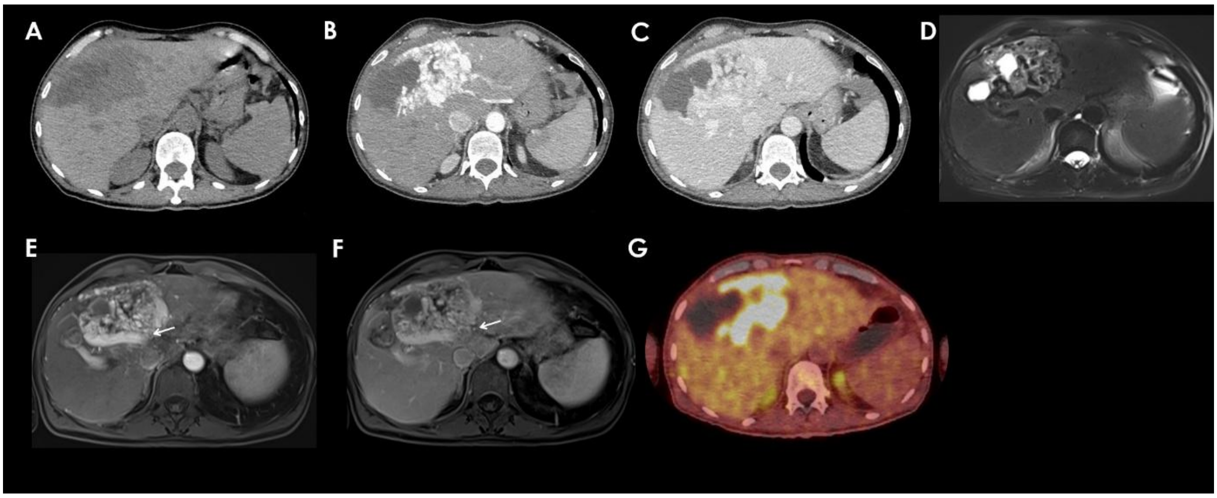

Supplementary Figure 4. Case of a renal PGL. 13 yo with SDHB.

The axial (A) and coronal (B) views of the enhanced CT Scan shows an early enhanced large retroperitoneal mass, exophytic heterogeneous, invading the anterior cortical the right kidney, with a solid and a cystic component. It measures 122 x 94 x 83 mm. We can see the washout on the portal phase axial (C) and coronal (D). It has an extension to the inferior vena cava. The differential diagnosis is renal cell carcinoma.

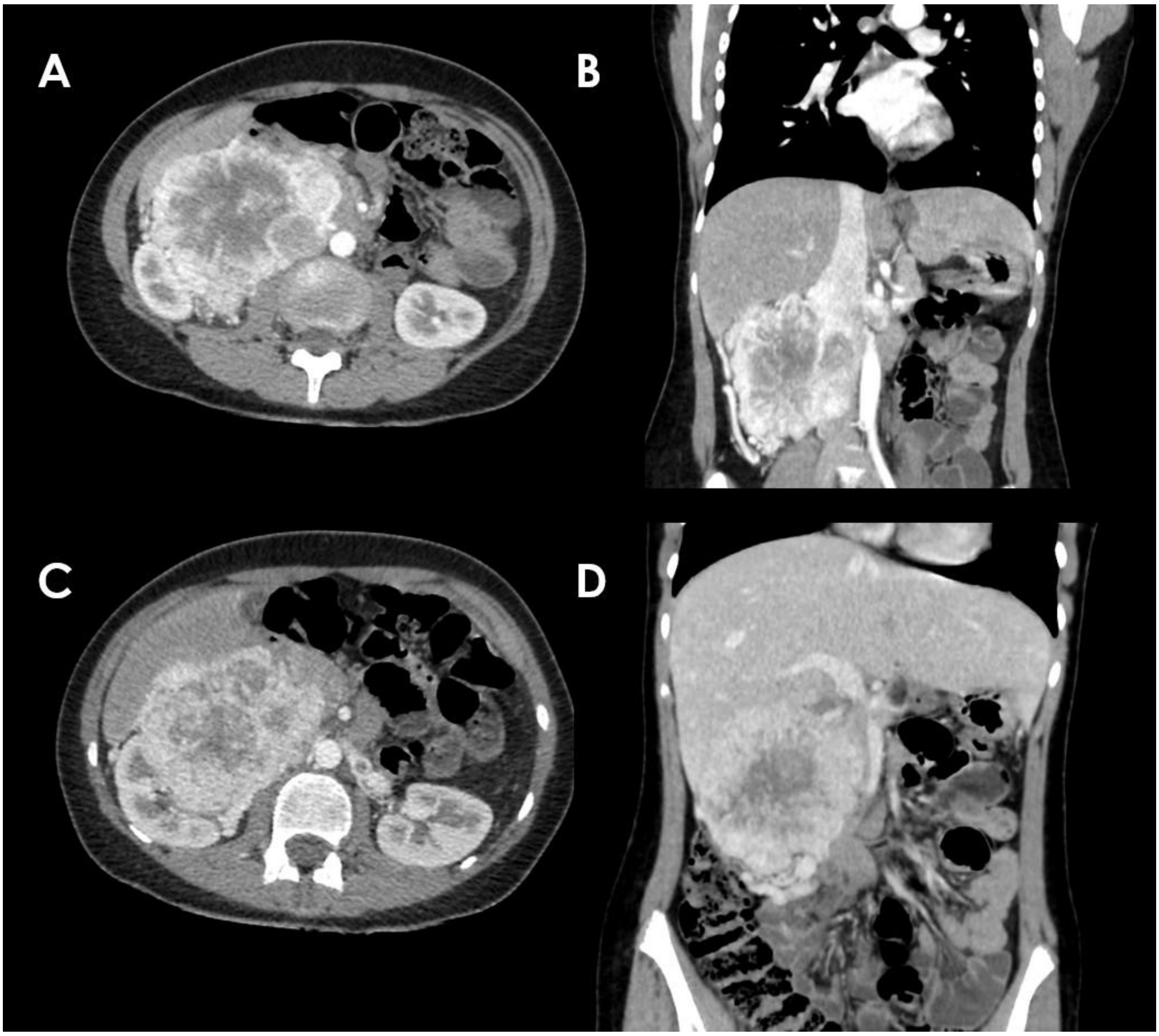

Supplementary Figure 5. Right PCC.

70 y.o. patient presenting a sporadic, functional PCC.

Heterogeneous adrenal mass presenting a peripheral enhancement on the coronal view of the injected CT Scan (A) and on T1-w CE MRI (B) with a central hypodense necrosis (A). It measures 69 x69 mm. There's a high uptake on ^{123}I-MIBG (SUVmax 9,8).

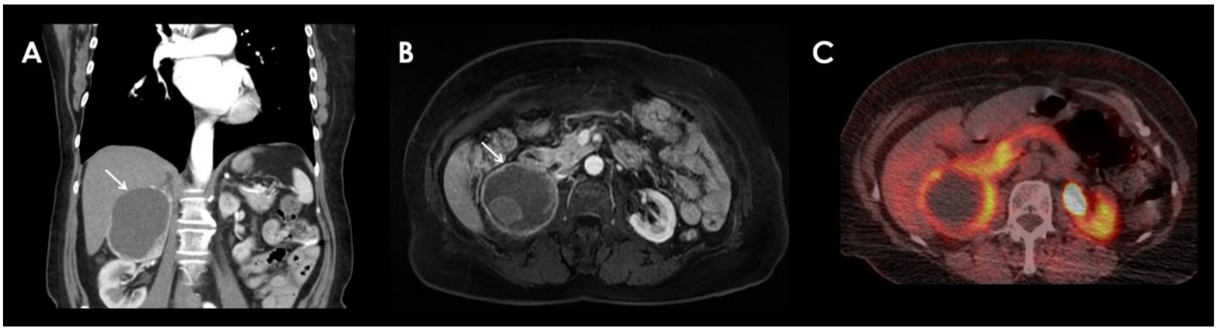

Supplementary Figure 6. Left PCC.

39 y.o. presenting an abdominal pain.

The non enhanced CT Scan shows the left adrenal mass (A), measuring 39 x 42 mm. An early enhancement is seen on the arterial phase axial (B). On the portal phase there's a de-enhancement (C).

There's a hyperfixation on the ^{18}F-FDG PET-CT SUV 14,6 (D).

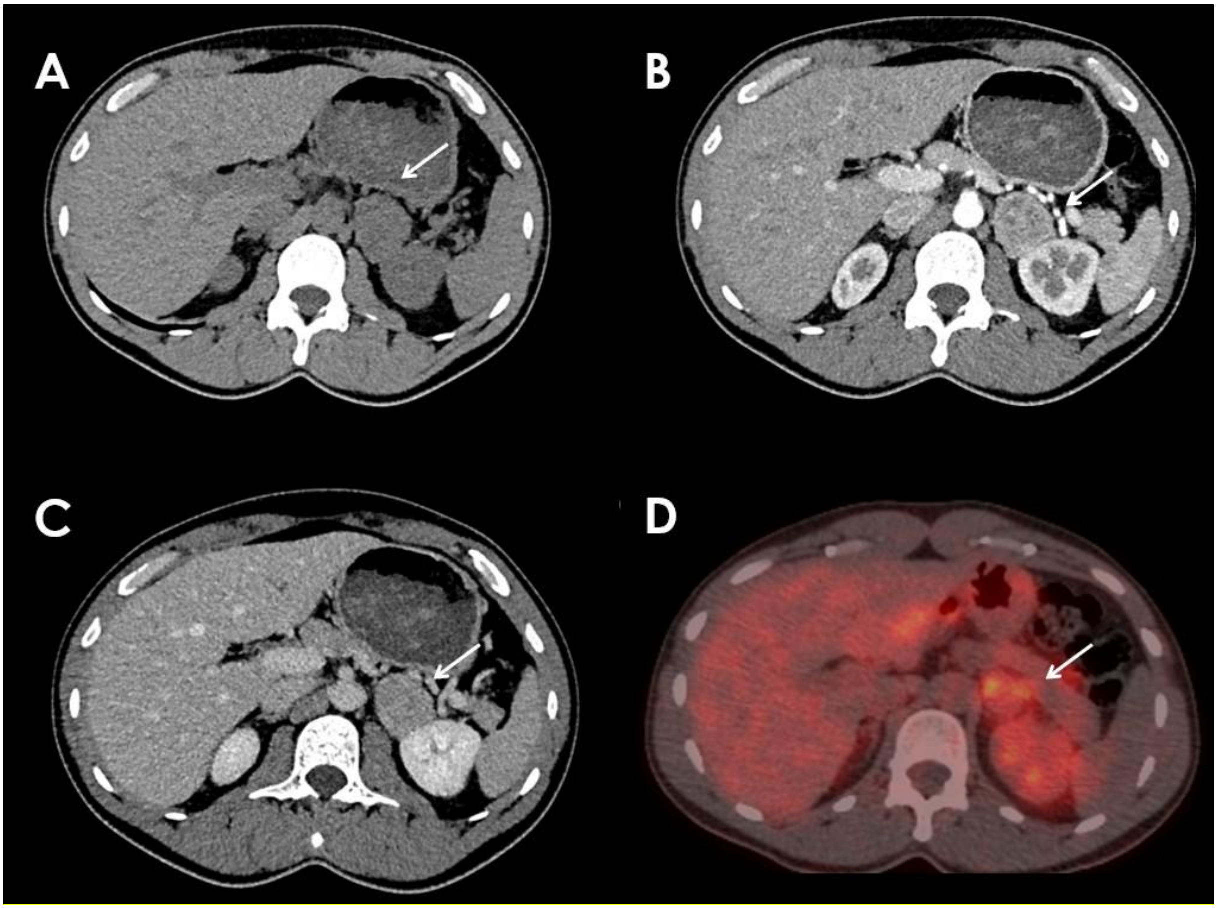

Supplementary Figure 7.

69 year old patient with penile squamous cell carcinoma (arrow) and initially suspected possible nodal metastasis (arrow), FDG avid on PET-CT, subsequently biopsied and consistent with PGL.

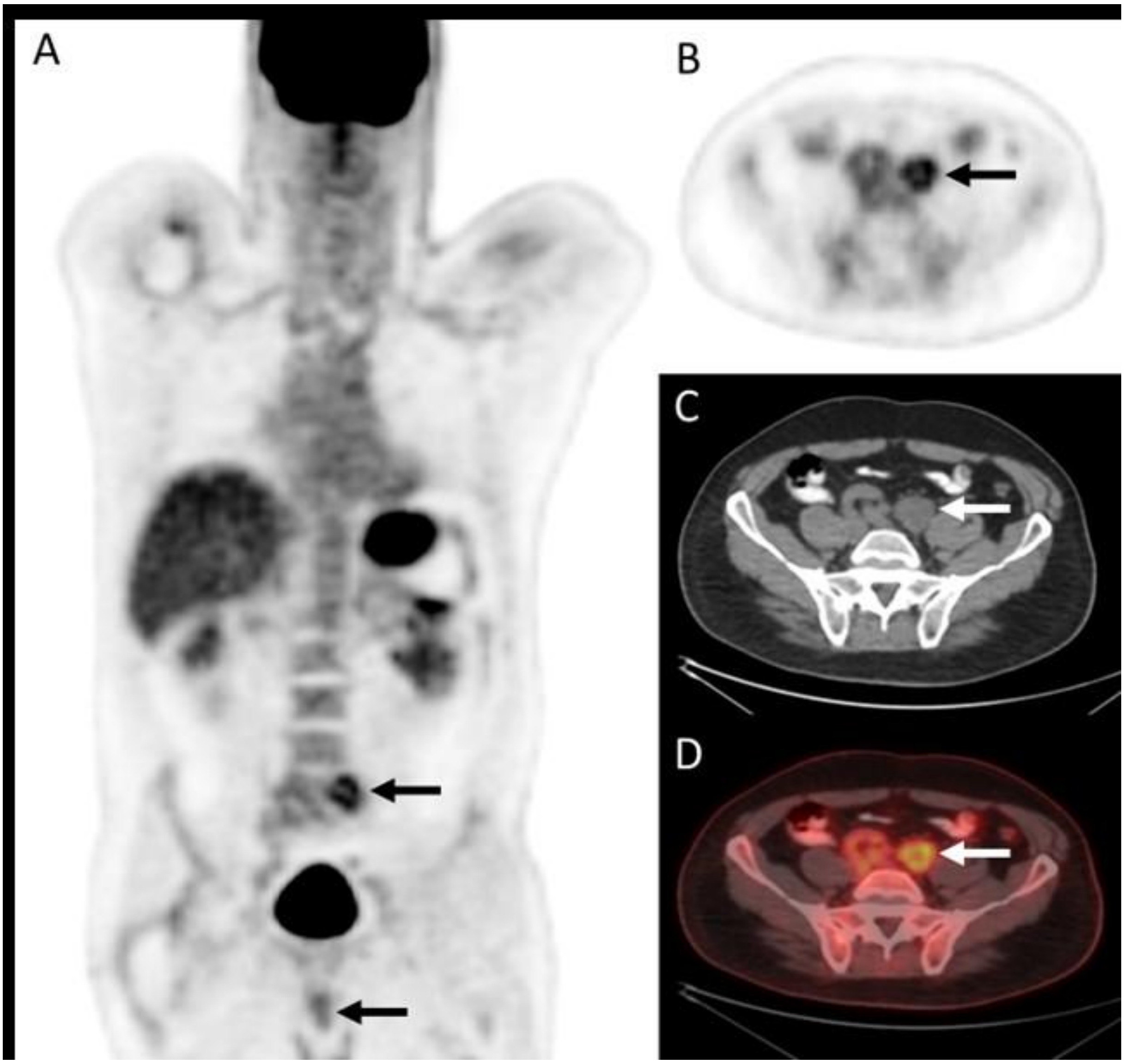

Supplementary Figure 8. Imaging findings of Right Jugulo-tympanic (non functional) PGL.

Axial iodine injected CT Scan (A), T1 Post Gadolinium Injection axial (B), showing the hypervascular lesion, measuring 22 x 20 mm. Tricks MRA sequence revealing the location of the PGL (D). See the Increased ^{18}F-FDG Uptake in the PET-CT (E), SUX max 36,8.

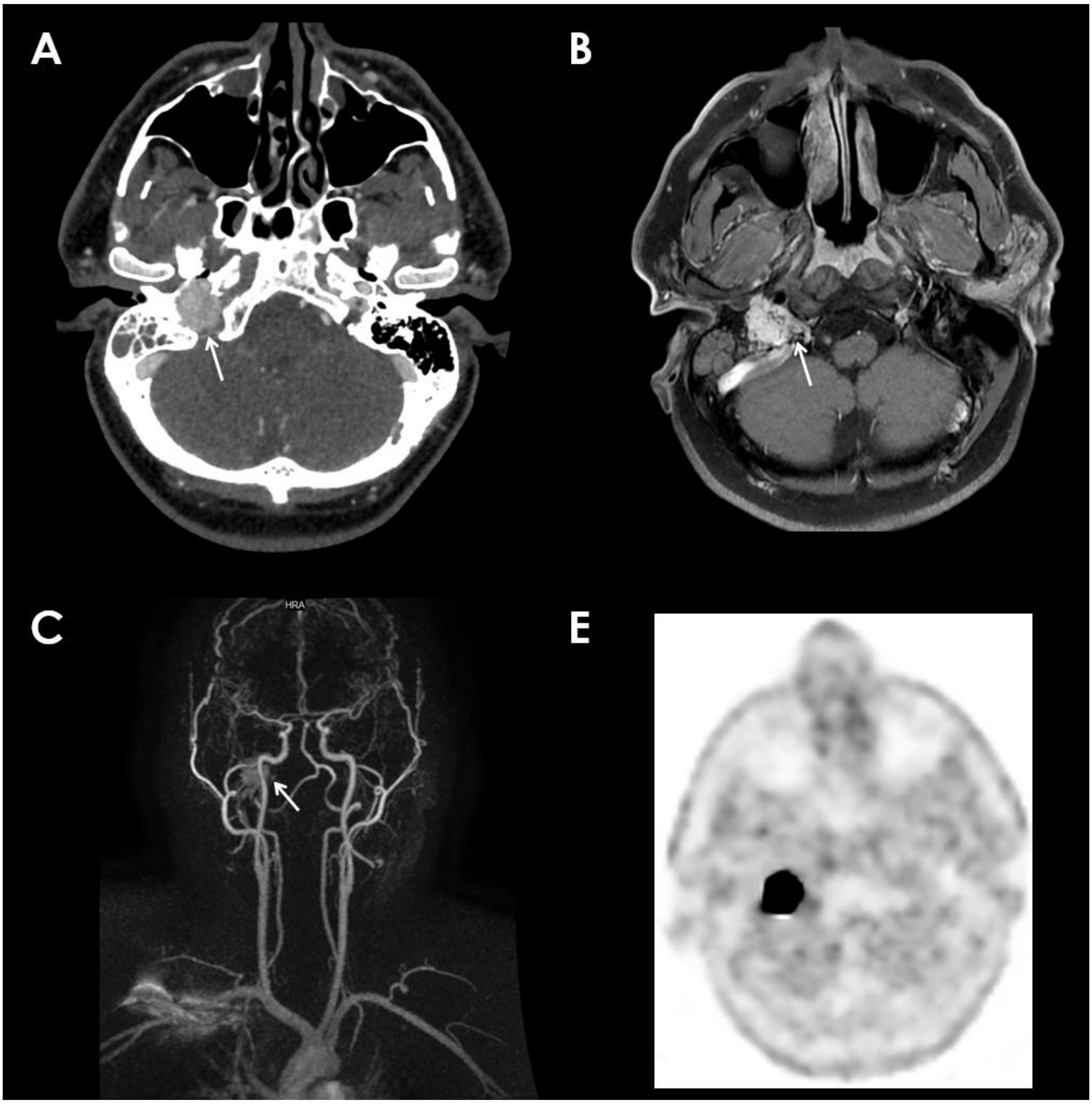

Supplementary Figure 9. Retroperitoneal PGL: Left latero aortic.

The coronal (A) and axial (B) views of the enhanced CT Scan shows an enhanced latero-aortic lesion. The MRI shows a well defined mass lying next to the abdominal aorta: coronal (C) and axial views (D) with heterogeneous enhancement on the T1-w CE sequence. High uptake at ^{18}F-DOPA PET-CT SUV max 14 (E). Differential Diagnosis are Castleman disease *(rare, non clonal lymphoproliferative disorder)* and lymph node fibrosis.

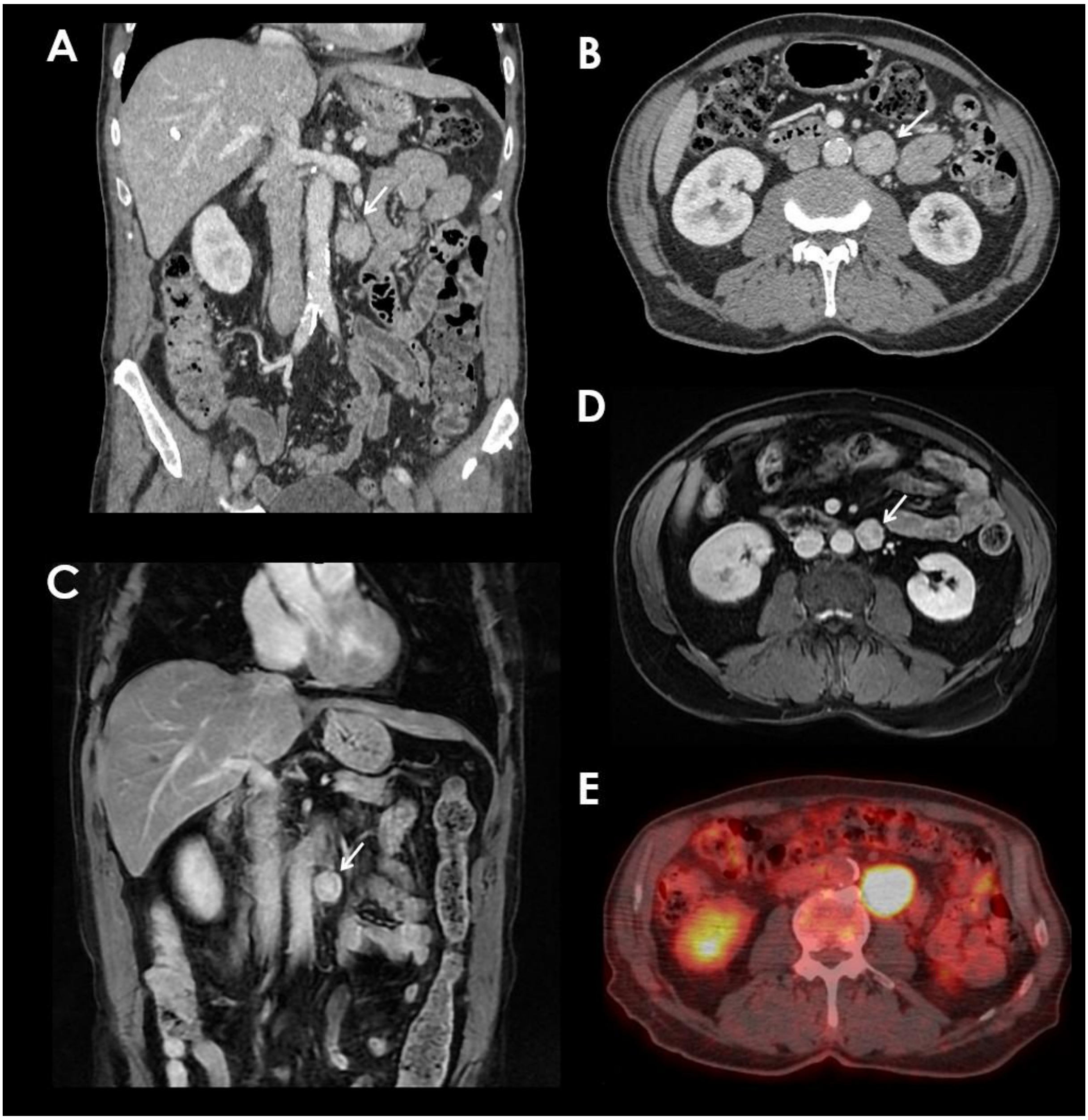

Supplementary Figure 10. Mesenteric PGL.

60 y.o. patient with a history of breast cancer with BRCA1 mutation, presenting a left superior mesenteric PLG, next to the mesenteric artery, seen on the axial and coronal views of the CT Scan (A-B), measuring 36 mm. There's a high uptake at ^{68}Ga-DOTA-SSTR PET/CT SUV max 9,3 (C), and at the ^{131}I-MIBG scintigraphy (D).

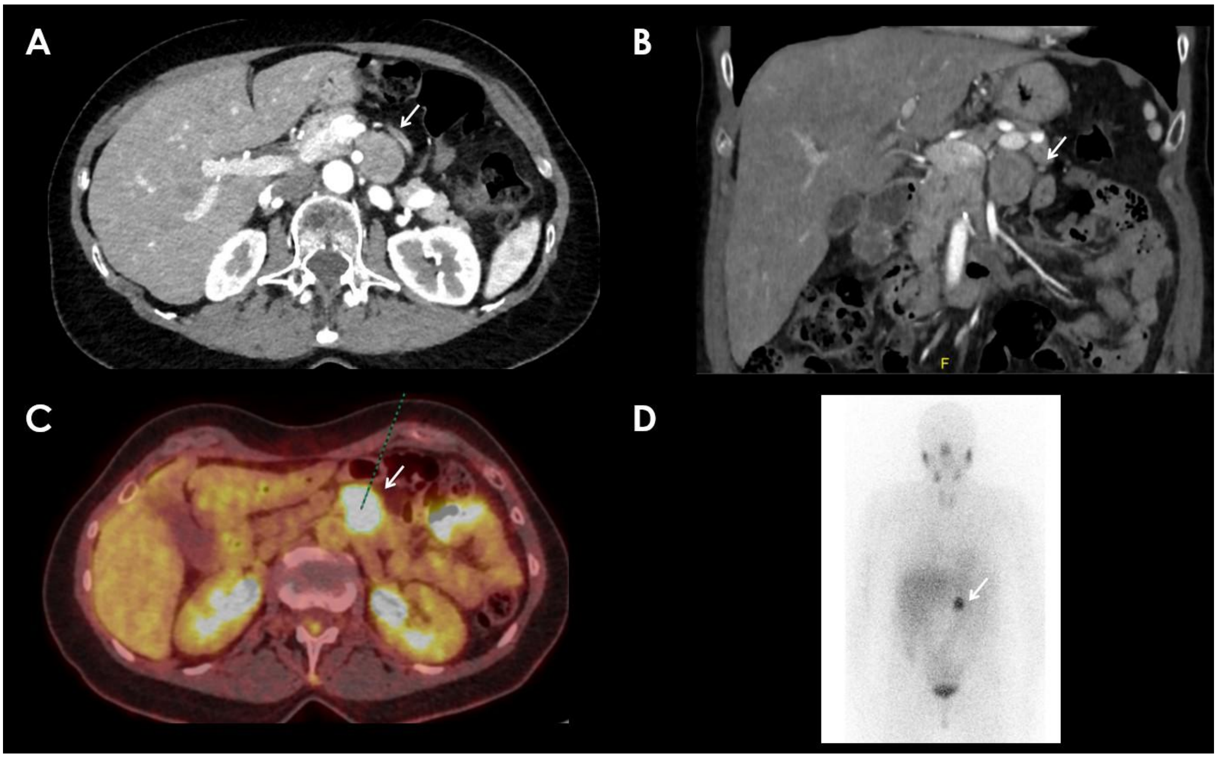

Supplementary Figure 11.

55 year old patient with bilateral carotid body PGLs visible on CT (D) and PET-CT (B-F). A mediastinal PGL (C, E, G) is also present, along with multiple hepatic and abdominopelvic nodal metastases, which show radiotracer avidity on ^{68}Ga-DOTA-SSTR PET-CT (A).

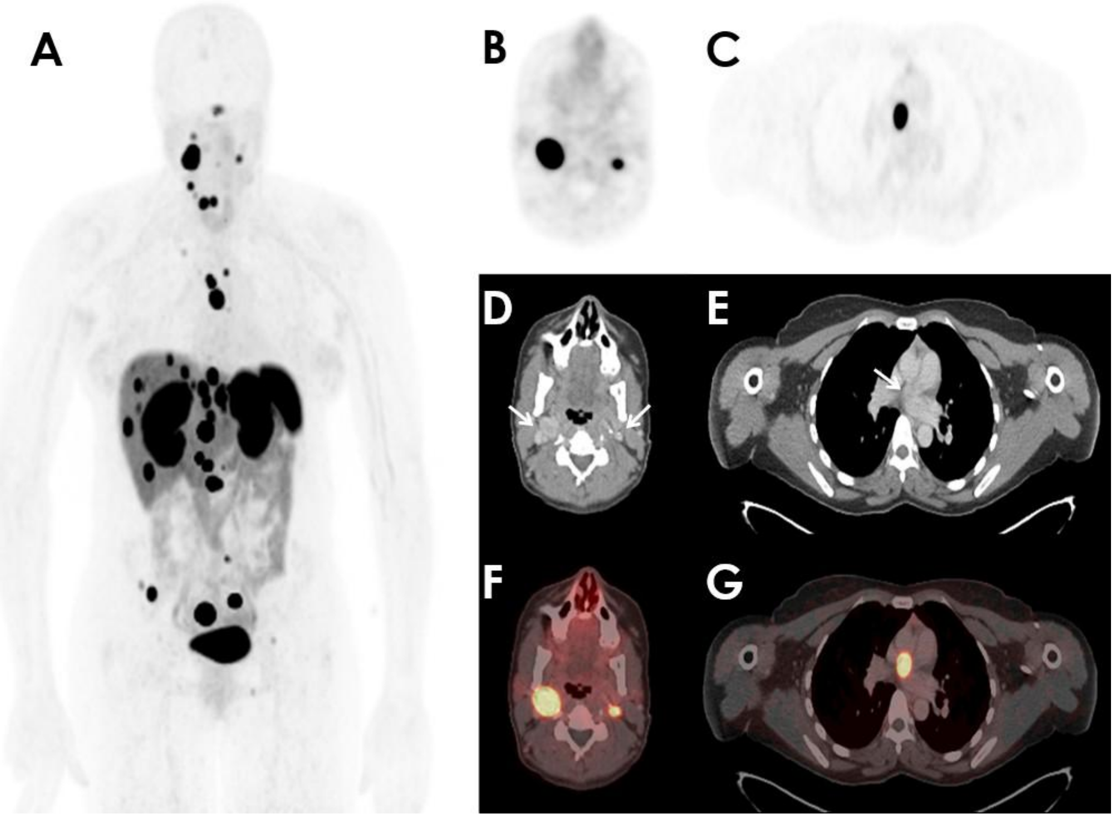

Supplementary Figure 12. Left Malignant PCC, SDHB mutation.

Large heterogeneous adrenal gland mass with cystic changes on the axial view of the non injected CT Scan (A), avidly enhancing specially in the periphery, on the arterial phase and portal phases (B and D respectively). In this case there's no calcifications within the PCC. We can see the downward displacement of the left kidney (B-D).

Mass effect is seen on the adjacent structures : the celiac trunk, the left adrenal gland, the left kidney, and the stomach. It measures 14 x 10 cm. A spleno-pancreatectomy and left nephrectomy was performed (E). High uptake at ^{18}F-DOPA PET-CT in dorsal spine (T12) SUV max 4,2 (C) and in the sacrum and in the ilia, SUV max à 14 (F).

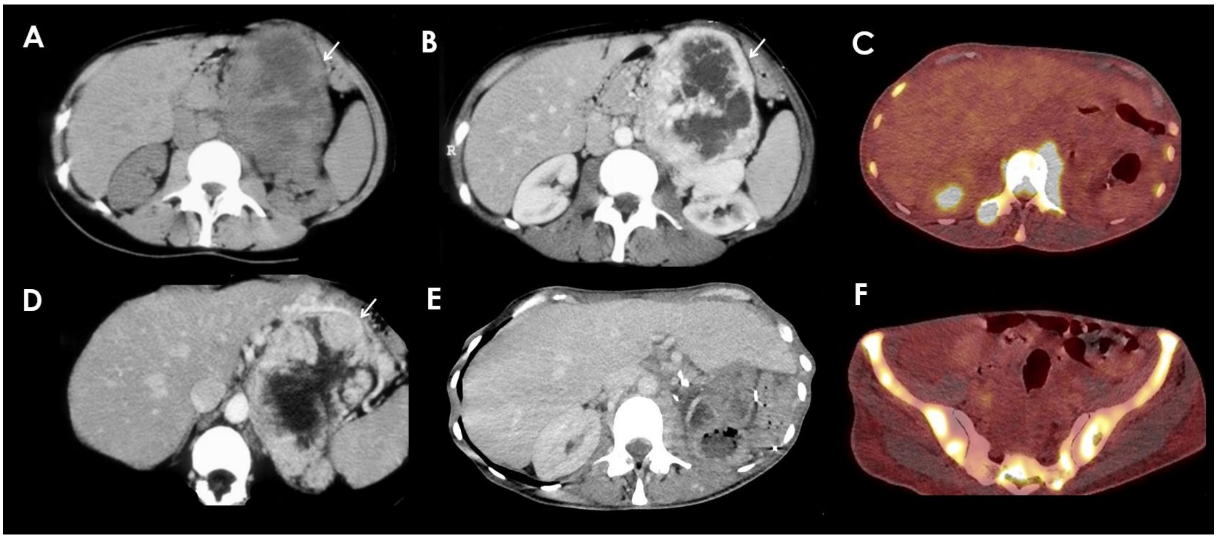

Supplement: Supplementary file 1 — ELECTRONIC SUPPLEMENTARY MATERIAL [file 13244_2025_2195_MOESM1_ESM.pdf]
